# Population screening for glucose-6-phosphate dehydrogenase deficiency using quantitative point-of-care tests: a systematic review

**DOI:** 10.3389/fgene.2023.1098828

**Published:** 2023-06-14

**Authors:** Mohamed Afiq Hidayat Zailani, Raja Zahratul Azma Raja Sabudin, Azlin Ithnin, Hafiza Alauddin, Siti Aishah Sulaiman, Endom Ismail, Ainoon Othman

**Affiliations:** ^1^Department of Pathology, Faculty of Medicine, Universiti Kebangsaan Malaysia (UKM), Kuala Lumpur, Malaysia; ^2^ UKM Medical Molecular Biology Institute (UMBI), Universiti Kebangsaan Malaysia Medical Centre, Kuala Lumpur, Malaysia; ^3^Department of Biological Sciences Dan Biotechnology, Faculty of Science and Technology, Universiti Kebangsaan Malaysia, Bangi, Malaysia; ^4^Department of Pathology, Faculty of Medicine and Health Sciences, Universiti Sains Islam Malaysia, Nilai, Malaysia

**Keywords:** glucose-6-phosphate dehydrogenase, point-of-care, quantita tive, screening, systematic review

## Abstract

**Background:** Glucose-6-phosphate dehydrogenase (G6PD) deficiency is an X-linked hereditary disorder and a global public health concern that is most prevalent in malaria-endemic regions including Asia, Africa, and the Mediterranean. G6PD-deficient individuals are at high risk of developing acute hemolytic anemia following treatment with antimalarial drugs including Primaquine and Tafenoquine. However, the currently available tests for G6PD screening are complex and often have been misclassifying cases, particularly for females with intermediate G6PD activity. The latest innovation of quantitative point-of-care (POC) tests for G6PD deficiency provides an opportunity to improve population screening and prevent hemolytic disorders when treating malaria. Aim(s): To assess the evidence on the type and performance of quantitative point-of-care (POC) tests for effective G6PD screening and hence, radical elimination of *Plasmodium* malaria infections.

**Methods:** Relevant studies published in English language confined from two databases, Scopus and ScienceDirect were searched from November 2016 onwards. The search was conducted using keywords including “glucosephosphate dehydrogenase” or “G6PD”, “point-of-care”, “screening” or “prevalence”, “biosensor” and “quantitative”. The review was reported following the PRISMA guidelines.

**Results:** Initial search results yielded 120 publications. After thorough screening and examination, a total of 7 studies met the inclusion criteria, and data were extracted in this review. Two types of quantitative POC tests were evaluated, namely, the CareStart^TM^ Biosensor kit and the STANDARD G6PD kit. Both tests showed promising performance with high sensitivity and specificity ranging mostly from 72% to 100% and 92%–100%, respectively. The positive and negative predictive values (PPV and NPV) ranged from 35% to 72% and 89%–100%, with accuracy ranging from 86% to 98%.

**Conclusion:** In areas with a high prevalence of G6PD deficiency that overlap with malaria endemicity, availability and validation of the diagnostic performance of quantitative POC tests are of absolute importance. Carestart™ biosensor and STANDARD G6PD kits showed high reliability and performed well in comparison to the spectrophotometric reference standard.

## 1 Introduction

Glucose-6-phosphate dehydrogenase (G6PD) deficiency is a genetic disorder that results in an inadequate amount of G6PD enzyme, a biological catalyst that is important to produce the reduced form of nicotinamide adenine dinucleotide phosphate that protects red blood cells against oxidative stress (Luzzatto et al., 2020). G6PD-deficient individuals are at high risk of developing acute hemolytic anemia and severe neonatal hyperbilirubinemia leading to brain damage upon exposure to exogenous agents, including certain food intakes such as fava beans, diseases such as bacterial infections, and drugs such as Primaquine and Tafenoquine (Chu et al., 2019; Lee et al., 2022). This inherited condition affects approximately 500 million individuals worldwide and is present most frequently in malaria-endemic regions such as parts of Asia, Africa, the Mediterranean, and the Middle East (Zeng et al., 2022). For instance, in Malaysia, a Southeast Asian country where malaria remains a serious public health concern due to its high forest coverage area, the prevalence of G6PD deficiency was 3.4%; among which 5.3% are males and 1.1% are females (Ainoon et al., 2003).

The disease is an X-linked hereditary disorder, meaning that it is most common in males. Phenotypically males can be either hemizygous G6PD deficient or normal, and females can be either homozygous or heterozygous G6PD deficient (Au et al., 2007). In heterozygote females, the range of G6PD activity is wide, ranging from completely deficient to perfectly normal activity levels. Normal G6PD activity is defined as the median G6PD activity level in subjects with no evidence of G6PD mutations. Based on the World Health Organization’s (WHO) latest recommendation, the current case definitions of G6PD deficiency are as follows: males and females with <30% of normal enzyme activity are G6PD deficient, heterozygous females with enzyme activity of 30%–80% are G6PD intermediate, and males with >30% and females with >80% activity levels are G6PD normal (World Health Organization, 2016).

Historically, a different classification was used back in the year 1985 which was known as the WHO G6PD Classification of Variants (Class I–V) (World Health Organization, 2019; He et al., 2020). However, there had been a gradual shift throughout the years from biochemical to mutation analysis in which it was later found that many G6PD variants which were classified as class II and class III have the same clinical manifestations (World Health Organization, 2016). Based on mutation analyses that were conducted in studies worldwide, most G6PD gene mutations (84%) are point mutations that affect a single nucleotide, while the remaining variants are multiple nucleotide substitutions, deletions, and intronic mutations. Different G6PD mutations produce a wide range of biochemical phenotypes such as decreased stability or decreased catalytic activity of the G6PD enzyme. In field application, there were high requirements to inform the G6PD status and the performance of point-of-care (POC) G6PD assays to guide clinicians on the treatment using antimalarial drugs such as Primaquine and Tafenoquine, which then have redefined a ‘normal’ G6PD activity to be either >70% or >80% of normal. Therefore, based on these findings, the G6PD classification was revised, modified, and updated as per the previously mentioned case definitions (World Health Organization, 2014).

The diagnosis of G6PD deficiency requires an appropriate and effective testing method, particularly for malarial patients who need to be treated with 8-aminoquinoline drugs including Primaquine and Tafenoquine. Malaria infection is caused by *Plasmodium* species including *P. ovale*, *P. vivax*, *P. falciparum*, *P. malariae*, and *P. knowlesi.* According to the WHO, the most common and lethal malaria parasite on the continent of Africa is *P. falciparum*, while the most common malaria parasite outside of sub-Saharan Africa is *P. vivax* (World Health Organization, 2023). Hypnozoites, which are liver-dormant stages, is a part of *P. ovale* and *P. vivax*’s life cycle. After the initial infection, these stages might reactivate and lead to malaria relapse in weeks, months, or even years. Relapses are substantially less common for *P. ovale* than for *P. vivax*. Nonetheless, to cure these patients, both the asexual stages of the parasites in the blood and the hypnozoites in the liver require to be eliminated–a process known as radical cure. Two important hypnozoiticidal agent that can be used for the radical cure of malaria and malaria chemoprophylaxis are Primaquine and Tafenoquine, which can cause severe hemolysis in individuals with G6PD deficiency (Commons et al., 2020).

Primaquine has a short half-life of 6 h and is rapidly metabolized and eliminated from the body. This medication can be prescribed to patients with ≥30% G6PD activity and thus can be prescribed based on a qualitative test result (Baird, 2018). However, a radical cure of malaria using Primaquine requires daily administration for 14 days and it is unclear that the Primaquine treatment is effective against the *P. ovale* hypnozoites due to limited studies. In contrast, Tafenoquine has a longer half-life of 14 days and can be prescribed as a single‐dose for radical cure of *P. vivax* and *P. ovale* malaria infection such as using the Kozenis (GlaxoSmithKline, Brentford, United Kingdom) 150 mg tablets or weekly regiments using the Kodatef 100 mg tablets (60 Degrees Pharmaceuticals, Washington, DC) for chemoprophylaxis. Both Primaquine and Tafenoquine should be discontinued early by the prescriber at the first signs of hemolysis. However, Tafenoquine discontinuation cannot reduce oxidative stress exposure in G6PD-deficient patients since the drug is removed slowly from the body with a lengthy half-life duration. As a result, the recommended G6PD activity threshold for Tafenoquine prescription is higher, and all females with moderate G6PD activity of less than 70% should be excluded (Dean et al., 2012). Hence, the safe use of Tafenoquine requires a precise quantitative test that can demonstrate G6PD activity within the normal G6PD reference range.

In addition, the strong link between G6PD deficiency and severe neonatal jaundice also led to the deployment of a national program of mandatory screening for G6PD deficiency in all infants since the early 1980s, using the WHO-recommended qualitative/semiquantitative Fluorescent Spot Test (FST) (Beutler and Mitchell, 1968). Although the method costs far less and was simpler to apply, it requires laboratory infrastructure and expertise in its interpretation. Additionally, studies found that there was a lack of sensitivity of the FST to detect G6PD-deficient individuals, particularly females with moderate enzyme activity ranging between 20% and 60% of the normal mean (Henriques et al., 2018; Thielemans et al., 2018). Henceforth, the condition necessitates the introduction of a quantitative method to measure and screen for G6PD deficiency among the population.

A gold standard diagnostic laboratory method at present is the quantitative ultraviolet (UV) spectrophotometry assay, which is complex, costly, and requires several hours of turn-around time, special equipment, a source of electricity, and experienced personnel. These various limiting factors make the spectrophotometry assay unsuitable for routine G6PD testing in most malaria-endemic countries and resource-limited settings (Pfeffer et al., 2020; Roper et al., 2020). Over the past few decades, all developed field applications for diagnosing G6PD deficiency including rapid diagnostic test (RDT) kit provide a qualitative result, which only reliably identifies whether the individual is normal or severely G6PD deficient with less than 30% residual enzyme activity (Ley et al., 2015). Although the tests were affordable, feasible, easily performed at the patient’s side, and better diagnostic than the conventional FST, its performance for G6PD carrier or G6PD deficient female heterozygotes remained poor and inaccurate in many cases (World Health Organization, 2015). The previous studies showed that most females with an intermediate enzyme activity level were often falsely classified as “normal” by the qualitative test (Yu et al., 2020; Djigo et al., 2021). These misclassifications were alarming as they increased the patients’ risk of hematological disorder upon administration of antimalarial drugs (Chu et al., 2018).

In addressing the diagnostic challenges and gender disparity issues by the currently available qualitative POC method, particularly for radical malaria elimination, the latest technological innovation of POC devices was developed to quantitatively measure G6PD enzyme activity. The assay was designed to be able to discriminate between the full range of deficiencies and therefore could accurately diagnose G6PD-deficient individuals. The novelty of the assay led to the aim of our current study which was to identify and assess available types of quantitative POC G6PD tests, compare the evaluation of performance from diverse laboratory and geographic contexts, and evaluate the strength and limitations of the quantitative assay.

## 2 Materials and methods

### 2.1 Study design

This is a systematic review of literature. This study was conducted and reported by following the principles of Preferred Reporting Items for Systematic Reviews and Meta-Analyses (PRISMA) guidelines (Supplementary Material 1) (Page et al., 2021).

### 2.2 Literature search strategy

The following electronic databases were searched from January 2017 to recent investigations in December 2021: Scopus and ScienceDirect, with restriction to English language publications. The last date of the database search was 26 December 2022. The keywords used were “glucosephosphate dehydrogenase”, “G6PD”, “point-of-care”, “screening”, “prevalence”, “biosensor”, and “quantitative”. The subject terms were combined using the Boolean operator “AND,” and “OR” for a comprehensive search. Additional relevant studies found from the references of selected articles were also retrieved and screened. An example of a search strategy for Scopus is as follows: TITLE-ABS-KEY [“glucose phosphate dehydrogenase deficiency” AND quantitative AND (“point-of-care” OR “biosensor”)].

### 2.3 Screening of articles for eligibility and quality assessment

Selected articles identified through the databases and references were screened for eligibility. The process started with a screening of the title and abstract and was followed by the selection of eligible studies that met the inclusion criteria developed from the research question using the PICOS (Population, Intervention, Comparator, Outcome, Study) component (Table 1). Exclusion criteria included studies that were performed using a qualitative type of point-of-care test for G6PD deficiency and irrelevant studies using other than cross-sectional methods such as economic evaluation and user perspective. Throughout the abstract screening, full-text articles were retrieved and read in the event of any uncertainty pertaining to the content relevance of the study. Once the list of abstracts was compiled and full articles were retrieved, all studies were evaluated by one researcher, while the second researcher compiled and reviewed the results. A third reviewer would be consulted if there was a disagreement on the research selection in order to come to a decision. The quality assessment and risk of bias in each study were assessed using the Appraisal tool for Cross-Sectional Studies (AXIS) (Downes et al., 2016).

**TABLE 1:** PICOS criteria for eligibility of studies.

Parameter	Inclusion criteria	Data extraction
Population	General population with Glucose-6-Phosphate Dehydrogenase (G6PD) deficiency with no age restriction	Location, Sample size
Intervention	Screening procedure using quantitative point-of-care test for G6PD deficiency	Prevalence of G6PD deficiency in studied population, mean and cut off thresholds of G6PD activity
Comparator	References spectrophotometric assay	Overall diagnostic performance of point-of-care test at different thresholds
Outcome	Quantitative identification of individuals with normal enzyme level and individuals with G6PD deficiency including severe and intermediate enzyme level	Sensitivity, specificity, positive predicitive value, and negative predictive value
Study	Cross-sectional/observational study	Study design

### 2.4 Data extraction

The following data was manually extracted from each study: name of the first author, year of publication, country or study location, study design, sample size, type of POC test, type of reference assay, results of the screening procedure including G6PD prevalence, levels of enzyme activity, and diagnostic performance of the POC tests. The extracted data was organized and collated into tables using Microsoft Excel Spreadsheet software.

### 2.5 Data synthesis

The tabulated data was then analyzed and interpreted by the first and second researchers. The primary outcomes assessed were the prevalence or the number of identified subjects with G6PD deficiency, the levels of G6PD activity for the studied population, the performance of the POC test including sensitivity, specificity, positive predictive value (PPV), and negative predictive value (NPV), and results of correlation analysis. This information was synthesized using a narrative (descriptive) method.

## 3 Results

### 3.1 Selection of articles

**FIGURE 1:**
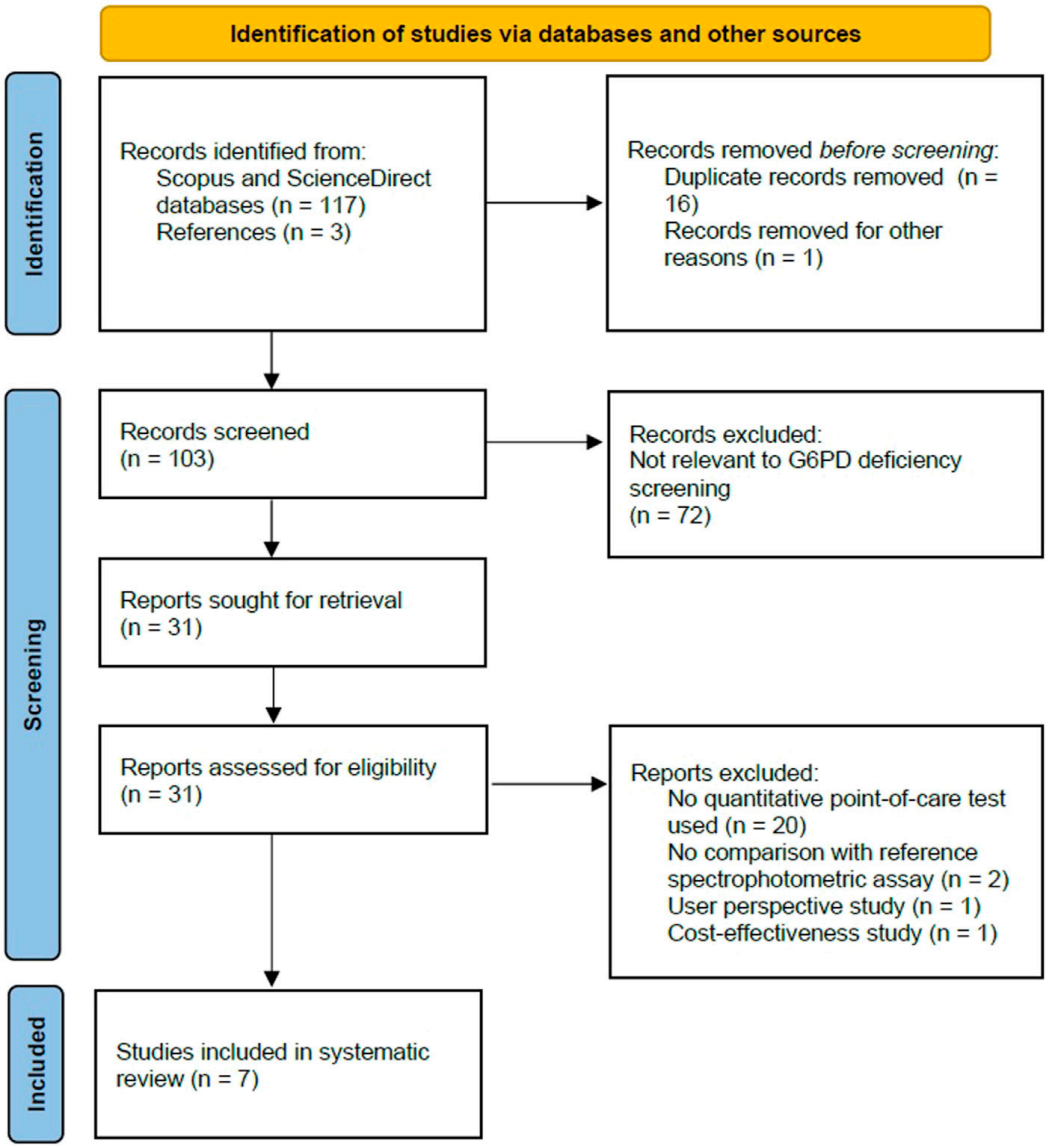
Flowchart showing inclusion in systematic review of studies reporting on diagnostic performance of quantitative point-of-care tests for screening of G6PD deficiency.

The selection process of articles for this study was summarized using the PRISMA flow diagram for systematic review (Figure 1). Overall, 117 publications were found during our initial search of the electronic databases. Three additional papers were found for this review from other sources, such as references from the searched articles. Following a duplicate removal, a total of 103 publications were screened for content relevance from the title and abstract. In case of doubt, the full-text versions of these articles were examined, and eligibility and full-text assessment were conducted on those that fulfill the inclusion criteria and were English publications. The final result of this study identified 7 articles that were included in this review, consisting of cross-sectional studies conducted from the year 2017 until 2021. An extracted data summarization from all studies was shown in Table 2.

**TABLE 2:** Summary of studies reviewing the diagnostic performance of the quantitative point-of-care test for screening of G6PD deficiency.

No.	First author and year of publication	Country	Title	Sample size *N*	Type of quantitative point-of-care (POC) test	References assay	Prevalence of G6PD deficiency in the studied population
1	Weppelmann et al. (2017)	Haiti	Field trial of the carestart biosensor analyzer for the determination of glucose-6-phosphate dehydrogenase activity in Haiti	343	CareStart™ G6PD Biosensor (CB; Cat. No. BGB-E00182; AccessBio, United States)	Trinity Biotech spectrophotometric assay (Kit No. 345B; Trinity Biotech, St. Louis, MO)	19.5% (67/343)
2	Ley et al. (2017)	Bangladesh	A comparison of three quantitative methods to estimate G6PD activity in the Chittagong Hill Tracts, Bangladesh	1,002	Carestart™ G6PD Biosensor (CB; AccessBio, United States)	Spectrophotometric assay kits (Randox,United Kingdom) on a Shimadzu UV 1800 (Kyoto, Japan)	9% (90/1002)
3	Alam et al. (2018)	Bangladesh	Field evaluation of quantitative point of care diagnostics to measure glucose-6-phosphate dehydrogenase activity	158	Carestart™ G6PD Biosensor (CB; AccessBio, United States) and STANDARD G6PD (SG; SDBiosensor, South Korea)	Shimadzu UV1800 spectrophotometric assay (Shimadzu, Kyoto, Japan)	43% (69/158)
4	Bancone et al. (2018)	Thailand and Myanmar	Validation of the quantitative point-of-care CareStart biosensor for assessment of G6PD activity in venous blood	150	CareStart™ G6PD Biosensor assay (CB; WellsBio, United States)	Trinity Biotech spectrophotometric assay (Trinity Biotech, Bray, Ireland)	66.7% (100/150)
5	Pengboon et al. (2019)	Thailand	Evaluation of quantitative biosensor for glucose-6-phosphate dehydrogenase activity detection	216	CareStart™ G6PD Biosensor (CB; WellsBio, Republic of Korea)	OSMMR2000-D (R&D Diagnostics, Ltd., Greece)	20.8% (45/216)
6	Pal et al. (2019)	United States of America (USA) and Thailand	Evaluation of a novel quantitative test for glucose-6-phosphate dehydrogenase deficiency: bringing quantitative testing for glucose-6 phosphate dehydrogenase deficiency closer to the patient	150	STANDARD™ G6PD (SG; SD Biosensor, South Korea)	Trinity Biotech spectrophotometric assay (Trinity Biotech, Bray, Ireland) and Pointe Scientific spectrophotometric assay, (Canton, MI; Cat No. G7583)	71.3% (107/150)
7	Zobrist et al. (2021)	Brazil	Evaluation of a point-of-care diagnostic to identify glucose-6-phosphate dehydrogenase deficiency in Brazil	1,736	STANDARD G6PD (SD Biosensor, Republic of Korea)	Pointe Scien-tific reagent kit (CatNo. G7583) and Shimadzu UV1800 spectrophoto-metric assay (Shimadzu, Japan)	5.4% (94/1736)

Level of G6PD activity by reference assay			Results of POC test diagnostic performance						Comments on POC test performance	Risk of bias
Mean (95% CI; IU/gHb)	Lower cut-off value (IU/gHb)	Upper cut-off value (IU/gHb)	Sensitivity (%)	Specificity (%)	Positive predictive value (PPV)	Negative predictive value (NPV)	Accuracy	Area under ROC curve (AUC)		
8.75	0.87 (10%)	5.24 (60%)	At 10% cut-off: 0.0	At 10% cut-off: 100	At 10% cut-off: Not available	At 10% cut-off: 98.8	At 10% cut-off: 98.8	*p* = 0.976	The CB consistently overestimated cases with moderate or severe G6PD deficiency and underestimated cases with normal G6PD activity	+
			At 30% cut-off: 5.9	At 30% cut-off: 99.7	At 30% cut-off: 66.7	At 30% cut-off: 90.6	At 30% cut-off: 90.4			
			At 60% cut-off: 53.7	At 60% cut-off: 94.6	At 60% cut-off: 70.6	At 60% cut-off: 89.4	At 60% cut-off: 86.6			
Not available (Adjusted Male Median: 7.03)	2.11 (10%)	4.22 (60%)	At 30% cut-off: 19	At 30% cut-off: 99	At 30% cut-off: 59	At 30% cut-off: 93	Not available	At 30%, *p* = 0.88	The CB showed a suboptimal performance and was less well correlated with the gold standard assay	++
9.90	2.97 (30%)	6.93 (70%)	1. For CB	1. For CB	Not available	Not available	Not available	1. For CB: *p* = 0.165	The CB showed a narrow value of clinical window for enzyme activity between 30% and 70%. The SG had a higher granularity and precision compared to the CB.	++
			At 30% cut-off: 72	At 30% cut-off: 100				2. For SG: *p* = 0.068		
			At 60% cut-off: 71	At 60% cut-off: 99						
			At 70% cut-off: 71	At 70% cut-off: 98						
			At 80% cut-off: 69	At 80% cut-off: 96						
			2. For SG	2. For SG						
			At 30% cut-off: 100	At 30% cut-off: 97						
			At 60% cut-off: 85	At 60% cut-off: 95						
			At 70% cut-off: 89	At 70% cut-off: 93						
			At 80% cut-off: 90	At 80% cut-off: 85						
7.91	2.37 (30%)	6.33 (80%)	At 30% cut-off: 100	At 30% cut-off: 92	Not available	Not available	Not available	*p* = 0.52	The difference between the ideal upper and lower cut-off values at 30% and 80% thresholds using the CB was narrow (1.55 IU/gHb)	++
			At 80% cut-off: 92	At 80% cut-off: 94						
7.30 (Adjusted Male Median: 8.1)	2.40 (30%)	6.50 (80%)	At 30% cut-off: 100	At 30% cut-off: 96	At 30% cut-off: 73	At 30% cut-off: 100	At 30% cut-off: 97	At 30% *p* = 0.982	The CB showed a high diagnostic ability at all thresholds	+
			At 70% cut off: 100	At 70% cut-off: 93	At 70% cut-off: 73	At 70% cut-off: 100	At 70% cut-off: 94	At 70% *p* = 0.993		
			At 80% cut off: 100	At 80% cut off: 91	At 80% cut-off: 73	At 80% cut off: 100	At 80% cut-off: 93	At 80% *p* = 0.978		
Not available (Adjusted Male Median: 8.89, 8.97, and 6.84)	2.7, 2.7, and 2.1 (30%)	7.1, 7.2, and 5.5 (80%)	At 30% cut-off: 100 for all sample types	At 30% cut-off: 97, 100, and 94.8	Not available	Not available	Not available	At 30% *p* = 0.99	The study highlighted the presence of a significant interlaboratory variability which impacts the ability to establish a perfect correlation between two assays	+
			At 70% cut-off: 95.5, 92, and 95	At 70% cut-off: 97, 80.5, and 81.6				At 70% *p* = 0.97		
			At 80% cut-off: 95, 97.8 and 96.3	At 80% cut-off: 86.3, 63.3 and 74.4				At 80% *p* = 0.97		
Not available (Adjusted Male Median: 8.9)	2.58, 2.67 (30%)	6.02, 6.23 (70%)	At 30% cut-off	At 30% cut-off	At 80% cut off: 35.2	At 80% cut-off	Not available	At 70% *p* = 1.0 (venous) *p* = 0.98 (capillary)	SG showed a good discriminatory capacity for G6PD-deficient individuals including females with intermediate G6PD activity	+
			100 (venous)	98.6 (venous)	99.8			At 80% *p* = 0.95 (venous) *p* = 0.90 (capillary)		
			100 (capillary)	97.8 (capillary)						
			At 70% cut-off	At 70% cut-off						
			96.9 (venous) 94.3 (capillary)	96.5 (venous) 92.3 (capillary)						
	TOTAL (*N*)			3,755						

### 3.2 Risk of bias

In general, all selected studies had a low to moderate risk of bias (Table 2). The studies applied an adequate approach to the research question and the findings were coherent in their sources, data collection, and analysis.

### 3.3 Main findings

This systematic review reports the evidence of the diagnostic performance of POC testing for G6PD deficiency across the globe, with a focus on a quantitative approach. A total of 3,605 individuals from seven different studies were universally screened for G6PD deficiency using quantitative POC tests. These studies demonstrated significant methodological diversity and statistical heterogeneity. It was notably found that the available type of quantitative POC tests was limited to two assay kits: CareStart™ Biosensor (CB) and STANDARD G6PD (SG). Four studies evaluated the CB, two studies evaluated the SG, and one study evaluated both CB and SG (Ley et al., 2017; Weppelmann et al., 2017; Alam et al., 2018; Bancone et al., 2018; Pal et al., 2019; Pengboon et al., 2019; Zobrist et al., 2021). Each study compared the POC diagnostic performance to a gold-standard spectrophotometric assay. They were conducted independently in six countries: Thailand, Myanmar, Bangladesh, Brazil, Haiti, and the United States (US).

According to the included articles, the prevalence of G6PD deficiency in the studied population ranged from 14.8% to 99.5%. Different lower and upper cut-off values were used in their evaluation including 10%, 30%, 60%, 70%, and 80% of normal enzyme activity. The 10% and 60% thresholds correspond to the WHO G6PD Classification of Variants’ Classes I, II, and III (World Health Organization, 2019). The 30% and 80% thresholds referred to the most recent WHO recommendation of the current case definitions of G6PD deficiency (World Health Organization, 2022), while the 70% threshold corresponded to the cut-off point for exclusion criteria of Tafenoquine treatment (Commons et al., 2020). For evaluation of the CB, the sensitivity, specificity, and positive and negative predictive values of the analyzer ranged from 0% to 100%, 91%–100%, 59%–73%, and 90.6%–100%, respectively. The corresponding values for the SG were 85%–100%, 74.4%–98.6%, 35.2%, and 99.8%. These results’ diversity reflected the variability in the study design and ethnography, as well as the rapid technological progress of the POC device over the years. Finally, the diagnostic accuracy was described only by two studies, which ranged from 86.6% to 98.8% (Weppelmann et al., 2017; Pengboon et al., 2019). All studies, except research conducted by (Alam et al., 2018; Bancone et al., 2018) reported excellent values of area under the receiver operating characteristic curve (AUC) which ranged from 0.88 to 1, denoting an outstanding overall diagnostic performance of the POC tests (Ley et al., 2017; Weppelmann et al., 2017; Pal et al., 2019; Pengboon et al., 2019; Zobrist et al., 2021).

## 4 Discussion

### 4.1 Summary of evidence

Many countries have promoted screening for G6PD deficiency in the general population, particularly in malaria-endemic areas and among newborns (Marasini et al., 2020). The most effective management strategy for malaria elimination and neonatal jaundice in G6PD-deficient neonates is early diagnosis of the hereditary condition. The informed G6PD status not only makes patients or the parents of affected newborns aware of the deficiency in order to avoid exposure to oxidative agents, but it also guides clinicians in the safe administration of medications such as antimalarial drugs and antibiotics. This systematic review expresses grave concern about the low reliability of currently available screening assays for G6PD deficiency, which is the FST, and advocates for the development of novel point-of-care diagnostics.

In contrast to the spectrophotometric assay, which requires trained personnel, a UV spectrophotometer, and other laboratory equipment, the POC devices were simple, compact, easily operated with minimal training, and convenient to be used in field application (Pal et al., 2019). One of the quantitative POC tests that are available in the market is the CareStartTM Biosensor (CB). Since 2017, five studies have been conducted to assess the robustness of this assay. This method requires a volume of 7–10 microliters (μL) of capillary or venous blood specimen, which could be directly applied to the analyzer’s strip. When the G6PD enzyme in the specimen combines with a substrate, this biosensor measures G6PD enzyme activity electrochemically by transferring electrons between donor and acceptor molecules. The magnitude of the generated electric current is directly proportional to the level of G6PD activity in the blood sample. Previous CB models, as used in studies by Weppelmann et al. (2017); Ley et al. (2017); Alam et al. (2018), could only quantify G6PD activity without hemoglobin analyses. The G6PD activity was later standardized to hemoglobin concentration using a separate digital hemoglobin meter or the results of a complete blood count analysis. Then, as used in studies by Bancone et al. (2018); Pengboon et al. (2019), a newer and more compact model of the CB test was developed which could perform both G6PD enzyme and Hb analyses in the same device.

In the earlier research, the CB performed poorly with a very low sensitivity of 5.9% at the 30% threshold for diagnosing moderate-to-severe G6PD deficiency (Weppelmann et al., 2017). It was also demonstrated that the CB only had a slight, or fair agreement between the assay and reference spectrophotometry in correctly determining the ordinal G6PD activity classification. The sensitivity of the CB was later proven to be higher in subsequent studies, which ranged between 19% and 72% using a similar biosensor model, indicating device technological improvement (Ley et al., 2017; Pengboon et al., 2019). In Alam et al. (2018) demonstrated that the CB performed well at 70% upper cut-off level with 71% sensitivity and 98% specificity, a threshold that is important for identifying heterozygous females and those contraindicated for tafenoquine treatment. Bancone et al. (2018) were the first to use the newer model of CB. The study revealed that the hemoglobin concentration measured by the new biosensor correlated well with the results from the complete blood count analysis of the study (*R* ^*2*^ = 0.88). The ranges of G6PD activities assessed through the study were almost identical between the CB and the gold standard spectrophotometric assay. The G6PD results of this study were found to be very similar in G6PD normal males but much higher in G6PD-deficient males and females. Despite the CB’s significant improvement over previous evaluations, the threshold points for distinguishing samples with activity ranging from 30% to 80% normal were demonstrated to be alarmingly narrow (Bancone et al., 2018). Further studies are warranted to investigate the small difference in “intermediate” G6PD activity between the two critical thresholds.

The STANDARD G6PD (SG) test is another quantitative POC G6PD assay that was designed to measure G6PD enzymatic level and total hemoglobin level simultaneously using the reflectometry assays method. Alam et al. (2018); Pal et al. (2019); Zobrist et al. (2021) used this test to evaluate its diagnostic performance in population screening. A total of 10 μL of capillary or venous blood specimen was required for this assay, which was mixed with a special extraction buffer solution provided by the manufacturer before being transferred to the designated device’s strip for biochemical detection. A study revealed that the correlation between the SG and spectrophotometric assay for both normalized G6PD activity and hemoglobin measurement was good, with squared correlation coefficient (*R* ^*2*^) values of 0.92 and 0.75, respectively (Pal et al., 2019). The strong and positive correlations of the test with high sensitivity ranged from 85% to 100% and specificity ranged from 74% to 100%, implying that it is a promising tool for screening for G6PD deficiency.

In addition, the SG performed well under a wide range of operating conditions, including temperatures ranging from 17°C to 43°C and humidity levels reaching 75% (Pal et al., 2019). This is significant because G6PD deficiency variants are widely distributed across malaria-endemic regions, including tropical Asian countries. According to the WHO prequalification technical specifications, an *in vitro* diagnostic test for G6PD deficiency must be able to distinguish between normal, G6PD-deficient with enzyme level below 30%, and G6PD-intermediate with 30%–80% of normal enzymatic activity (World Health Organization, 2016). Current findings proved that the SG’s performance was excellent at the 30% threshold and acceptable at the 70% and 80% thresholds.

It is important to note that each of the seven studies included in this systematic review had its own set of limitations. Small to moderate sample sizes, potential selection bias, and lack of diversity among participants were among the few limitations. For example, Weppelmann et al. (2017) highlighted the use of convenience sampling from a single Haiti’s department in their study design which resulted in findings that might not be representative of another department or the whole country, and Pengboon et al. (2019) discovered that the diagnostic efficacy of the CB was higher in their study than in previous evaluation studies, which could be attributed to their small sample size (n = 216). The limitation was also emphasized by Zobrist et al. (2021), who described that there were a lower-than-expected number of participants with deficient and intermediate G6PD status in their study, which subsequently reflected in the sensitivity and specificity values at 95% confidence intervals.

Another limitation is that most evaluation studies were performed in laboratory settings with highly regulated environmental temperature and humidity. This limitation was highlighted by Bancone et al. (2018); Pal et al. (2019), who suggested that future clinical studies be conducted in near-patient or intended settings in order to be more reflective of the intended use and essential to further validate the device’s performance and robustness. Nonetheless, all reported studies in this systematic review have considered the distribution and genotypic-phenotypic map of G6PD deficiency in their study designs. This correlation is normally demonstrated in chronic nonspherocytic hemolytic anemia (CNSHA) G6PD-deficient individuals, which present in individuals with Class I G6PD variants. However, most G6PD mutations worldwide are Class II and II, and these mutations show ethnogeographic variability with a specific spectrum of variants in different ethnicities. As an example, a study by Koromina et al. (2021) revealed the large extent of variability in mutations of G6PD deficiency across worldwide population and highlight its population-specific genetic composition.

Similarly, the studies included in this review to evaluate the performance of the quantitative device were performed across multiple study sites with a high prevalence of G6PD deficiency and carefully selected specific ethnic groups such as the Afro-Haitian population in the Republic of Haiti by Weppelmann et al. (2017), Tibeto-Asian and Bengali descent in Chittagong Hill Tracts, Bangladesh by Ley et al. (2017); Alam et al. (2018), Burman and Karen ethnic groups in northwestern Thailand by Bancone et al. (2018); Pengboon et al. (2019); Pal et al. (2019), and Manaus and Porto Velho population in Brazil by Zobrist et al. (2021).

### 4.2 CareStart^TM^ Biosensor (CB) versus STANDARD G6PD (SG) tests

Only a study conducted by Alam et al. (2018) provides a direct comparison of both POC tests. The study was performed in Bangladesh, and the sample size was 158 participants. Both devices demonstrated a high correlation with the reference spectrophotometric assay and performed well under field and laboratory conditions with comparable accuracy. At a 30% cut-off, the AUC for both tests did not differ significantly, indicating that they have comparable discriminatory power. However, at 70% cut-off activity, only the SG achieved high sensitivity, reaching to 90%, while the sensitivity of the CB was 70%. According to this study, the CB had a smaller difference in the clinically relevant window between 30% and 70% G6PD activity than the SG, with values ranging from 4.6 to 6.8 IU/gHb and 2.5–6.4 IU/gHb, respectively. This slightly wider window suggested that the SG had more granularity than the CB.

### 4.3 Strengths and limitations

The strength of our study is that it is a systematic review that looks at novel quantitative point-of-care testing for population screening for G6PD deficiency. It is useful in guiding policymaking in relation to screening for hereditary conditions, prescribing important medications, and combating malaria infection. One limitation of this review is the heterogeneity of the studies retrieved. Although all studies included are cross-sectional studies, high methodological diversity in their evaluation of device performance was expected with different populations and laboratory settings. Apart from that, we did not prospectively register the protocol of this study in any international database due to concerns about the expected major delays in checking and publishing registrations (Puljak, 2021).

## 5 Conclusion

This review looks at the outcome of quantitative point-of-care testing methods for G6PD deficiency in terms of their diagnostic performance, strength, and limitation. Given the high prevalence of G6PD deficiency in certain regions of the world that overlap with malaria endemicity, effective point-of-care diagnostics may provide enhanced safety to all affected individuals in these areas including males, females, and neonates, improve management of *P. vivax, P. ovale*, and *P. falciparum* malaria cases in resource-limited settings, and avoid drug resistance evolution. The CB and the SG demonstrated high reliability as a POC test, and further research into their effectiveness and feasibility should be conducted before real-life clinical implementation in health facilities.

## Data Availability

The original contributions presented in the study are included in the article/supplementary material, further inquiries can be directed to the corresponding author.
